# Retinol and α‐tocopherol in pregnancy: Establishment of reference intervals and associations with CBC

**DOI:** 10.1111/mcn.12975

**Published:** 2020-03-05

**Authors:** Jingrui Liu, Sien Zhan, Yan Jia, Youran Li, Ying Liu, Ying Dong, Guodong Tang, Lin Li, Yanhong Zhai, Zheng Cao

**Affiliations:** ^1^ Department of Laboratory Medicine Beijing Obstetrics and Gynecology Hospital, Capital Medical University Beijing China; ^2^ Prenatal Diagnosis Center Beijing Haidian Maternal and Child Health Hospital Beijing China; ^3^ Central Laboratory Beijing Obstetrics and Gynecology Hospital, Capital Medical University Beijing China

**Keywords:** anaemia, Hoffmann method, inflammation, LC‐MS/MS, maternal nutrition, nutritional status, pregnancy, quantitative methods, reference interval, retinol, α‐tocopherol

## Abstract

Profound physiological changes during pregnancy may affect the requirement of retinol and tocopherol, which are essential micronutrients for the maintenance of maternal health and foetal development. However, the current reference intervals (RIs) of retinol and tocopherol are based on non‐pregnant population. In the present study, a liquid chromatography–tandem mass spectrometry quantitation method for serum retinol and α‐tocopherol was established and validated. In addition, we established trimester‐specific RIs of retinol and α‐tocopherol using the data from paired screening test for 31,301 outpatients who participated in the prenatal vitamins A/E evaluation program at our hospital using the Hoffmann method, which is a simple indirect RI estimation method that does not require the recruitment of healthy subjects. Further, to explore the associations between the levels of retinol and α‐tocopherol and the parameters of complete blood count (CBC), the results of retinol, α‐tocopherol, and CBC of 1,977 pregnant outpatients in the third trimester were analysed. The testing interval between the levels of vitamins and CBC was no more than 7 days. Although no significant changes were noticed in the levels of retinol, the α‐tocopherol levels continuously increased with normal physiological changes throughout pregnancy. Lower retinol levels were associated with the higher incidence of anaemia, whereas higher levels of retinol and lower levels of α‐tocopherol were associated with higher platelet count.

**Key messages**

- The retinol level of the pregnant population in our study showed no significant changes throughout the pregnancy, while the α‐tocopherol level was increased significantly with the increase in gestational age.
- High level of retinol can reduce the risk of anemia during pregnancy.
- Increased retinol and decreased α‐tocopherol were associated with higher platelet counts, implying their different roles in inflammation.
- A simple quantitation method for serum retinol and α‐tocopherol by LC‐MS/MS was established and validated with good performance.

## 1 INTRODUCTION

Retinol and tocopherol are essential micronutrients for the maintenance of maternal health and foetal development. Retinol facilitates the normal functioning of visual system, immunity, reproduction, cell growth, and differentiation. It is also instrumental in promoting lung development and maturation of the developing foetus (Graham‐Maar, Schall, Stettler, Zemel, & Stallings, 2006). Tocopherol is a potent antioxidant, which scavenges free oxidative radicals in human body and plays a vital role in regulating cell proliferation and gene expression (Raizman et al., 2014).

Profound physiological changes occur during pregnancy, which may affect the requirement of retinol and tocopherol in pregnant women. The need for retinol is normally increased throughout the gestation period because of the physiological increase in blood volume and foetal development during this period (Mcguire, 2012). Pregnant women tend to be more sensitive to the adverse effects of tocopherol deficiency, including weakness, fatigue, dermatitis, creatinuria, reduced thyroid function, and increased bleeding than non‐pregnant adults (Rumbold, Ota, Hori, Miyazaki, & Crowther, 2015). Tocopherol has been reported to augment the effect of anticoagulants such as warfarin, though the underlying mechanisms remain unclear (Rumbold et al., 2015).

Liquid chromatography–tandem mass spectrometry (LC‐MS/MS) has been widely used in clinical laboratories for quantitation of vitamins. It offers better analytical specificity as compared with immunoassays and has higher throughput than gas chromatography–mass spectrometry (Grebe & Singh, 2011). LC‐MS/MS needs less sampling volume and simpler pre‐analytical process than high‐performance liquid chromatography (Furr, 2004). However, despite its advantages over other techniques, only a few reports about LC‐MS/MS assays for simultaneous determination of retinol and tocopherol in serum have been published (Albahrani, Rotarou, Roche, & Greaves, 2016). Therefore, one of the aims of the present study was to establish and validate an efficient and reliable LC‐MS/MS method for simultaneous quantitation of retinol and α‐tocopherol in serum.

Recently, there has been a growing interest in the importance of retinol and tocopherol estimation and supplementation during pregnancy (Chen, Li, Mao, Kang, & Zhang, 2018; Chen, Qian, Yan, & Jiang, 2018). However, the current recommended reference intervals (RIs) of retinol and tocopherol are based on non‐pregnant population (Bastos Maia et al., 2018; West, 2002) and whether they are suitable for pregnant women is questionable. Therefore, the need of the hour is to evaluate and establish trimester‐specific RIs for retinol and tocopherol for the correct interpretation of results and appropriate clinical intervention.

The traditional RI‐establishing method requires the recruitment of at least 120 healthy subjects (Horowitz et al., 2010), which is laborious and time‐consuming. A problem here is that there is nobody as “absolute healthy,” and it is very difficult for laboratory professionals to rule out all the subclinical diseases or the abnormalities that may affect the value of target analyte (Katayev, Fleming, Luo, Fisher, & Sharp, 2015). The challenge is further magnified while targeting different age groups and uncommon sample types (Katayev, Balciza, & Seccombe, 2010).

The Hoffmann method was first described in 1963 and is an indirect method for RI estimation that does not require the recruitment of healthy subjects (Hoffmann, 1963). The present study was aimed to estimate the RIs of retinol and α‐tocopherol by the Hoffmann method using the existing laboratory data from a relatively large patient group (the screening data from outpatients), wherein the retinol and α‐tocopherol levels were within the normal limits in most of the subjects. Katayev et al. (2010, 2015) showed the reliability of Hoffmann approach using a computer program and optimizing it with new functions and algorithms using data‐mining techniques (Katayev et al., 2010; Katayev et al., 2015). Later, the professional groups such as International Federation of Clinical Chemistry Committee on Reference Intervals and Decision Limits highlighted the importance of indirect techniques for RI estimation (Jones et al., 2018). In the present study, we aimed to calculate the trimester‐specific RIs of retinol and α‐tocopherol in pregnant women using the Hoffmann method. We also evaluated the associations between the retinol and α‐tocopherol levels during pregnancy and the complete blood count (CBC) measurements in the outpatients who participated in the prenatal vitamin evaluation program at our institute.Key Messages 1‐The retinol level of the pregnant population in our study showed no significant changes throughout the pregnancy, while the α‐tocopherol level was increased significantly with the increase in gestational age. 2‐High level of retinol can reduce the risk of anemia during pregnancy. 3‐Increased retinol and decreased α‐tocopherol were associated with higher platelet counts, implying their different roles in inflammation. 4‐A simple quantitation method for serum retinol and α‐tocopherol by LC‐MS/MS was established and validated with good performance.

## 2 METHODS

### 2.1 Subjects

Totally, 31,301 outpatient pregnant women visiting the obstetrics department at our institute from July 2016 to June 2017 participated in the vitamins A and E assessment program, which is routinely recommended by the obstetricians for all the registered pregnant women. The vitamin measurements were used for the subsequent estimation of RIs. Among all the participants, 11,429 were in the first trimester (1–13 gestational weeks), 10,214 in the second trimester (14–27 gestational weeks), and 9,658 in the third trimester (28–39 gestational weeks) of pregnancy.

For the association studies, 1,977 pregnant outpatients whose levels for vitamins A, E, and CBC were estimated in the third trimester were recruited from July 2017 to June 2018. The time interval between vitamins test and CBC test was no more than 7 days.

### 2.2 LC‐MS/MS method for quantitation of retinol and α‐tocopherol in serum

#### 2.2.1 Chemicals and reagents

The certified reference standards (retinol and α‐tocopherol) and isotope‐labelled internal standard (IS) solutions (d6‐retinol and d6‐α‐tocopherol) were purchased from Cerilliant Corporation (Round Rock, TX, USA). The methanol, acetonitrile, and formic acid (LC/MS grade) were obtained from Fisher Scientific (Fair Lawn, NJ, USA). The deionized water was purchased from Watsons (Hong Kong, China).

#### 2.2.2 Preparation of calibrators and quality controls

The stock solutions of retinol (12.4 μg/ml) and α‐tocopherol (1 mg/ml) were diluted with 6% bovine serum albumin solution (Procell, Wuhan, China) to prepare the 6‐point calibrators of the following concentrations (retinol, α‐tocopherol): Calibrator 6 (2 μg/ml, 40 μg/ml), Calibrator 5 (1 μg/ml, 20 μg/ml), Calibrator 4 (0.5 μg/ml, 10 μg/ml), Calibrator 3 (0.25 μg/ml, 5 μg/ml), Calibrator 2 (0.1 μg/ml, 2 μg/ml), and Calibrator 1 (0.06 μg/ml, 1.2 μg/ml). High‐level quality control solution (1.2 μg/ml retinol, 24 μg/ml α‐tocopherol) and low‐level quality control solution (0.2 μg/ml retinol, 4 μg/ml α‐tocopherol) were similarly prepared from the stock solutions after diluting with 6% bovine serum albumin. The IS mixture was prepared with methanol, acetonitrile, and water (1:1:2).

#### 2.2.3 Instrumentation and conditions

The assay presented in this study for simultaneous quantitation of serum retinol and α‐tocopherol was modified from the previously reported in‐house LC‐MS/MS method (Jia et al., 2018). The method development and validation were done on Triple Quad™ 5500 mass spectrometer by Sciex (Framingham, MA, USA) coupled with a high‐performance liquid chromatography system (LC‐20ADXR) by Shimadzu (Kyoto, Japan).

Briefly, using a Venusil MP C18 column (Agela Technologies, Tianjin, China) and the elution system comprising 0.02% formic acid in water (Mobile Phase A) and 0.1% formic acid in methanol (Mobile Phase B), the chromatographic separation of retinol and α‐tocopherol was completed in 3 min at a flow rate of 0.9 ml/min.

The analytes were detected by the mass spectrometer with scheduled multiple reaction monitoring in the positive electrospray ionization mode. The MS instrumental settings have been described previously (Jia et al., 2018). The following primary (quantitative) and secondary (qualitative) ion pairs were used: m/z 269.1/119.2 and 269.1/213.2 for retinol; m/z 431.3/165.2 and 431.3/137.0 for α‐tocopherol. For the IS, the following ion pairs were used: m/z 275.4/96.2 for d6‐retinol and m/z 437.3/171.2 for d6‐α‐tocopherol. All data were collected using the Sciex Analyst software (Sciex, Framingham, MA, USA) and analysed using the MultiQuant 2.1 software (Sciex, Framingham, MA, USA).

#### 2.2.4 Sample preparation

The whole blood samples were collected and centrifuged at 3,000 rpm for 10 min to obtain the sera, which were kept at 4C in dark before use. A highly efficient one‐step sample preparation was done, wherein for each serum sample, 300‐μl internal standard, 50‐μl zinc sulfate solution (2 M), and 20‐μl serum/QC/calibrator were mixed in a 96‐well protein precipitation plate equipped with a collecting plate. The flow‐through eluent obtained under a pressure of 3–6 psi was further diluted with 50% methanol before the LC‐MS/MS analysis.

#### 2.2.5 Assay validation

The method was validated for linearity, limit of detection (LOD), limit of quantitation (LOQ), precision, accuracy, and sample stability. Specifically, the linearity was evaluated using the 6‐point calibrators by measuring the ratio of analyte peak area to IS area against the nominal concentrations. A correlation coefficient (*R*) of.99 or higher was considered acceptable. The LOD and LOQ were determined by analysing the serially diluted QC specimens spiked with IS over 3 days of period. The LOD was defined as the average concentration obtained after three consecutive measurements with signal to noise (S/N) ratio >3 and coefficient of variation (CV) <20%. The LOQ was defined as the average concentration obtained after three consecutive measurements with S/N ratio >10 and CV <20%. The stability of the analytes in serum was assessed by evaluating serum samples kept at 4°C for up to 7 days. The accuracy was calculated as the percentage of the recovered analyte concentration to its nominal concentration (0.5 and 2 μg/ml for retinol, 10 and 40 μg/ml for α‐tocopherol). The intra‐assay imprecision was estimated by analysing the controls for 10 times in the same run. The interassay imprecision was estimated by analysing the controls once a day for a total of 20 days.

### 2.3 Complete blood count analysis

The routine complete blood count (CBC) analysis was performed on the Sysmex XN‐2000/3000 automatic blood cell analyzer (Sysmex Corporation, Kobe, Japan) following the standard operation procedure recommended by the manufacturer.

### 2.4 Establishment of RIs using the Hoffmann method

The detailed steps for the estimation of RIs can be found in Hoffmann's article published in 2016 (Hoffmann, Lichtinghagen, & Wosniok, 2016). Briefly, the data were entered in an MS Excel spreadsheet (Microsoft Corporation, WA, USA). The results of vitamin tests for a specific trimester were logarithmized and then sorted in an ascending order. Further, the formula = NORM.INV was applied to generate a new set of distribution numbers to create a quantile–quantile plot (Q–Q plot) with the log‐transformed data. Furthermore, the linear portion of the Q–Q plot was visually examined, and the Y values were calculated when X was equal to −2 and 2, using the best‐fit linear regression equation. With a standard normal distribution, the interval between μ ± 2σ represents the central 95% range of the measurements or the RI (μ represents the mean and σ represents the standard deviation), as defined by the Clinical and Laboratory Standards Institute guidelines (Horowitz et al., 2010). The lower and upper limits of the estimated RIs were calculated by antilogarithmizing the Y values from the previous step. The Q–Q plot for retinol is shown in Figure S1.

### 2.5 Association between retinol and α‐tocopherol levels and the CBC parameters

Using the trimester‐specific RIs obtained in the present study, the patients in the association study were divided into the vitamin deficient group and the sufficient group. The anaemia in pregnancy was diagnosed when a patient's haemoglobin concentration was lower than 110 g/L (Achebe & Gafter‐Gvili, 2017). The Mann–Whitney *U* test was used to examine statistical differences in the levels of vitamins among women in three trimesters of pregnancy. The SPSS 21.0 software (IBM Corporation, New York, USA) was used for all statistical analyses. The χ^2^ test was used to compare the prevalence of anaemia in the groups stratified by vitamin status. The Mann–Whitney *U* test was used to compare the levels of CBC parameters including red blood cell count, mean corpuscular volume (MCV), mean corpuscular haemoglobin (MCH), haematocrit (HCT), mean corpuscular haemoglobin concentration, red cell distribution width, platelet count (PLT), and white blood cell count (WBC). The probability value *p* < .05 in a two‐sided test was considered as statistically significant.

### 2.6 Ethical considerations

This study was approved by the Ethics Committee of BJOGH. The written informed consents were decided to be exempt from the participants as this was a retrospective study with the data collected from the routine vitamin evaluation program provided for our outpatients.

## 3 RESULTS

### 3.1 Assay validation for LC‐MS/MS method

As summarized in Table 1, the linear range of the method was 0.06–2 μg/ml for retinol and 1.2–40 μg/ml for α‐tocopherol. The correlation coefficient (*R*) for retinol and α‐tocopherol was.998 and.999, respectively. The LOD and LOQ of retinol was 0.02 and 0.03 μg/ml, respectively; whereas that of α‐tocopherol was 0.01 and 0.8 μg/ml, respectively (Table 1). The analytes were found to be stable in serum for at least 7 days, when stored at 4°C. The recovery rates and reproducibility (measured by both intra‐assay and interassay imprecision) were also satisfactory (Table 1).

**Table 1:** Validation of LC‐MS/MS method for retinol and α‐tocopherol quantitation

Validation parameter	Retinol	α‐Tocopherol
*R*	.998	.999
Linear range (μg/ml)	0.06–2.00	1.2–40.0
LOD (μg/ml)	0.02	0.01
LOQ (μg/ml)	0.03	0.8
Stability (%)	102	89
Accuracy (%)	107–108	89–92
Intra‐assay QC‐L CV (%)	3.1	5.2
Intra‐assay QC‐H CV (%)	2.0	2.8
Interassay QC‐L CV (%)	3.7	5.3
Interassay QC‐H CV (%)	4.9	6.7

Abbreviations: CV, coefficient of variation; LC‐MS/MS, liquid chromatography–tandem mass spectrometry; LOD, limit of detection; LOQ, limit of quantitation; QC‐H, high‐level quality control; QC‐L, low‐level quality control; R, correlation coefficient.

### 3.2 Trimester‐specific RIs for retinol and α‐tocopherol

**Figure 1:**
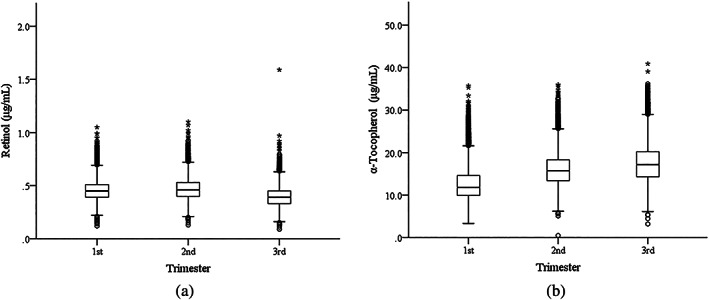
The box plots of retinol and α‐tocopherol levels during the three trimesters in pregnancy. The lower and upper horizontal lines of the boxes define the 1.5 interquartile range (IQR) of the lower quartile and the 1.5 IQR of the upper quartile, respectively. The wide horizontal lines mark the median values. The outliers are defined as the data outside of the following range (Q1−1.5 IQR, Q3 + 1.5 IQR). They are presented as circles or asterisks that are below or above the horizontal bars. The horizontal lines outside the boxes represent the minimum or maximum values. (a) retinol levels in three trimesters; (b) α‐tocopherol levels in three trimesters

The levels of both retinol and α‐tocopherol displayed statistically significant difference among different trimesters in pregnant outpatients (Mann–Whitney *U* test, *p* < .001). Specifically, compared with those in the first and second trimesters, the retinol level decreased in the third trimester, whereas α‐tocopherol level showed a continuous elevation throughout the pregnancy (Figure 1). The trimester‐specific RIs for retinol and α‐tocopherol, shown in Table 2, were established using the Hoffmann method.

**Table 2:** Reference intervals of retinol and α‐tocopherol in three trimesters by Hoffmann method

	1st trimester	2nd trimester	3rd trimester	Nonpregnancy^a^
Retinol (μg/ml)	0.3–0.7	0.3–0.7	0.3–0.6	0.3–0.8
α‐Tocopherol (μg/ml)	7–21	10–24	11–28	5–18

a The nonpregnancy reference intervals were adopted from Tietz textbook (see Connell, 2012).

### 3.3 Association between retinol and α‐tocopherol levels and CBC

The association study demonstrated a significant effect of retinol levels on the prevalence of anaemia. The rate of anaemia was 14.8% in the retinol sufficient group, whereas it was increased to 23.4% in the retinol deficient group, thereby suggesting that retinol deficiency may be a risk factor for anaemia during pregnancy (Table 3). Further, the rate of anaemia in α‐tocopherol sufficient group (15.6%) was lower than that of α‐tocopherol deficient group (22.9%), but the difference was not statistically significant.

**Table 3:** Associations between the levels of retinol and α‐tocopherol and anaemia

	Number of anaemia/total (%)	*p* value
Retinol deficient	49/209 (23.4)	.001
Retinol sufficient	262/1768 (14.8)	
α‐Tocopherol deficient	11/48 (22.9)	.166
α‐Tocopherol sufficient	300/1929 (15.6)	

*Note.* The association data were collected from the third trimester pregnant women. Retinol deficient: <0.3 μg/ml; retinol sufficient: ≥0.3 μg/ml; α‐tocopherol deficient: <11 μg/ml; α‐tocopherol sufficient: 11 μg/ml.

Interestingly, the averages of WBC and PLT count in the retinol sufficient group were higher than those in the retinol deficient group, whereas the average PLT count of α‐tocopherol sufficient group was lower than that of the α‐tocopherol deficient group. Additionally, both retinol sufficient group and α‐tocopherol sufficient group showed slightly higher averages of MCV and MCH than those in the corresponding deficient groups. No significant association was found between retinol and α‐tocopherol levels and the rest parameters of CBC (Table 4).

**Table 4:** Associations between the levels of retinol and α‐tocopherol and complete blood count parameters

	Significant difference with CBC parameters	Deficiency group (median)	Sufficiency group (median)	*p* value	No statistical difference with CBC parameters
Retinol	PLT (10^9/L)	192	201	.009	RBC, RDW, HCT, MCHC
	WBC (10^9/L)	8.47	9.13	.001	
	MCV (fl)	89.7	91.1	<.001	
	MCH (pg)	30.8	31.2	.002	
α‐Tocopherol	PLT (10^9/L)	219	199	.002	WBC, RBC, HCT, MCHC, RDW
	MCV (fl)	89.5	91.0	.037	
	MCH (pg)	30.5	31.2	.042	

Abbreviations: CBC, complete blood count; HCT, haematocrit; MCH, mean corpuscular haemoglobin; MCHC, mean corpuscular haemoglobin concentration; MCV, mean corpuscular volume; PLT, platelet count; RBC, red blood cell count; RDW, red cell distribution width; WBC, white blood cell count.

## 4 DISCUSSION

The in‐house developed LC‐MS/MS method for serum retinol and α‐tocopherol quantitation had good linearity, precision, and accuracy. In contrast with other previously reported MS methods measuring retinol and α‐tocopherol, the present method has the advantages of simple sample preparation, shorter chromatographic run time, and better precision and accuracy (Midttun et al., 2016). More importantly, the assay, as a laboratory‐developed test provided for our patients, complied with the quality requirements of the College of American Pathologists by passing its external proficiency testing of retinol and α‐tocopherol quantitation in 2018 (data not shown).

The modified Hoffmann method adopted in the present study neither requires the recruitment of healthy people nor sophisticated computer data processing for determining RIs. Moreover, the entire method can be completed in the MS Excel software and therefore, may potentially have wide applications in clinical laboratories.

Although the application of the Hoffmann method in RI estimation has increasingly drawn attention, some reports of incorrect implementation have also emerged. An article by Holmes and Buhr (2019) showed that using an empirical cumulative distribution function plot in a linear scale (with an alpha set at.05), rather than a normal Q–Q plot, resulted in artificially narrowed RIs (Holmes & Buhr, 2019). The method we applied in the present study is the modified and simplified form of the “normal Q–Q plot” validated by Georg Hoffmann in 2016 (Hoffmann et al., 2016). In particular, our method converted the Hoffmann's cumulative probabilities plot into a Q–Q plot so that the RIs could be calculated from the linear regression equations. The superiority of Q–Q plot over probability plot lies in that it makes the graph shape a strictly straight line, ensuring the accuracy of the results obtained from the simulated linear equation.

The RIs currently being referred to are 0.3–0.8 μg/ml for retinol and 5–18 μg/ml for α‐tocopherol (Connell, 2012). They are mainly based on the consensus or the guidelines from epidemic surveys on healthy adults rather than pregnant women. The majority of the participants in the present study were apparently healthy women in different pregnancy stages. They had sufficient levels of retinol and α‐tocopherol according to the RIs derived from non‐pregnant population. This suggests that our study cohort was appropriate for RI establishment with the Hoffmann method. However, whether the RIs derived from the statistical Hoffmann method are clinically more appropriate still needs to be further evaluated in future studies involving both healthy and vitamin deficient pregnant women.

The fact that the trimester‐specific RIs for retinol were essentially comparable with the RIs for non‐pregnant women suggested that retinol levels did not vary significantly during pregnancy. In contrast, the trimester‐specific RIs for α‐tocopherol by the Hoffmann method showed an overt elevation with the increase in gestational age. Chen, Qian, et al. (2018) conducted a similar investigation on vitamins A, D, and E in pregnant women, although it was not stratified by trimesters. Chen's study including 1,056 pregnant Chinese women showed that the lower limit (12.7 μg/ml) of 95% confidence interval for vitamin E level was much higher than that for the non‐pregnant women (5 μg/ml). Additionally, the rate of deficiency was negligible (0.19%). Therefore, whether the existing RIs for vitamin E, based on non‐pregnant women, can fulfil the maternal need to support the development of the growing baby may be questionable.

Regarding retinol, the RIs of the three trimesters by the Hoffmann method were essentially similar to the RIs for non‐pregnant women. Although the RIs of the first and second trimesters were identical, upper limit of the RI in the third trimester was slightly decreased compared with those in the previous two trimesters. Similarly, Yang's study on Chinese women showed that with the increase in gestational period, the levels of retinol declined (Yang et al., 2016). Additionally, a guideline on retinol supplementation in pregnant women pointed that the increased requirement for retinol in pregnant women is mainly limited to the third trimester (Mcguire, 2012).

Owing to the lack of universally accepted clinical guidelines focusing on the retinol and tocopherol supplementation during pregnancy, general advice on dietary and vitamin supplementation has been usually offered by clinicians to women with vitamin deficiency. Therefore, the establishment of trimester‐specific RIs for retinol and tocopherol will provide good reference and solid evidence for the clinicians who aim to evaluate the nutritional status of pregnant women. For instance, the present study demonstrated that the RIs for α‐tocopherol showed a steady increase with the increasing gestational age, thereby suggesting that the basic requirement for α‐tocopherol during pregnancy continuously increased. In other words, the α‐tocopherol deficient patients may be overlooked while referring to the RIs based on non‐pregnant women. We also examined the associations between retinol and α‐tocopherol levels and anaemia. A strong negative correlation was observed between retinol level and the incidence of anaemia, with the prevalence of 14.8% in retinol sufficient group and 23.4% in retinol deficient group. Our results are consistent with the previous reports on retinol levels and anaemia (Semba & Bloem, 2002; Wang et al., 2016). A previous report suggests that retinol supplementation may enhance erythropoietin expression and iron mobilization for erythropoiesis in order to improve haemoglobin level (da Cunha, Campos Hankins, & Arruda, 2018). However, no significant association was observed between α‐tocopherol level and anaemia.

Further, the CBC results showed that the PLT count was higher in retinol sufficient group, whereas it was lower in α‐tocopherol sufficient group. Platelets have been reported to be acute phase reactants in inflammation (Strukova, 2006). Retinol insufficiency has been reported to impair immune responses to infection and epithelial barrier function, resulting in the increased susceptibility to immunologic defects and inflammatory disorders (Rubin, Ross, Stephensen, Bohn, & Tanumihardjo, 2017). A study published decades ago reported that retinol induces platelet aggregation via activation of phospholipase A2 (Nakano, Hanasaki, Matsumoto, & Arita, 1988). The results of another study showed that there was no significant long‐term effect of retinol supplementation on the PLT count (Cartmel, Moon, & Levine, 1999). Therefore, more studies are required to elucidate the association between retinol levels and platelet counts during pregnancy.

We found that the higher α‐tocopherol levels were associated with lower PLT count, which is consistent with the results of Wintin's study (Whitin, Gordon, Corwin, & Simons, 1982). A number of studies have shown that α‐tocopherol could inhibit the platelet functions, including activation, aggregation, and adhesion, thereby contributing to their protection mechanism for cardiovascular disease (Freedman & Keaney, 2001; Murohara et al., 2002). Two major forms of tocopherol have been reported in human body, α‐tocopherol and γ‐tocopherol, which differ by the number and position of methyl groups on the chromanol ring (Wagner, Kamal‐Eldin, & Elmadfa, 2004). Both of the forms may have opposite associations with inflammation. A previous study based on lungs has shown that in contrast to the anti‐inflammation effect of α‐tocopherol, γ‐tocopherol was beneficial for the neutrophilic inflammation by reacting with reactive nitrogen species (Marchese et al., 2014). In addition, it has been shown that γ‐tocopherol, which is a major diet form for vitamin E in the United States, displayed complementary effects to those of α‐tocopherol in protection against chronic diseases (Wagner et al., 2004). The high ratio of α‐tocopherol to γ‐tocopherol had been suggested as an important discriminator between subjects with coronary heart disease and controls (Ohrvall, Sundlof, & Vessby, 1996). In a study conducted in five European countries, the ratio of α‐tocopherol to γ‐tocopherol showed differences among countries and was defined as the marker related to the healthy or protective effects of the Mediterranean‐like diet (Olmedilla et al., 2001). In the present study, only α‐tocopherol levels were measured, and it could not provide the accurate estimation of the antioxidants level of vitamin E. In the future, it would be better to measure the ratio of α‐tocopherol to γ‐tocopherol to understand the relationship between tocopherol and inflammation.

Additionally, the retinol sufficient group presented a higher WBC count than the deficient group, thereby implying that retinol may participate in the upregulation of immune function and inflammation (Brown & Noelle, 2015). Both retinol sufficient group and α‐tocopherol sufficient group showed slightly higher averages of MCV and MCH than the corresponding deficient groups in our study. Interestingly, Zhang, Ni, and Hu (2018) showed similar positive correlation between MCH and retinol levels (Zhang, Ni, & Hu, 2018). Tocopherol has been shown to prevent cell membrane from being damaged by reactive oxygen species to maintain red blood cell integrity and increase the number of erythroid colony forming units in bone marrow (Jilani & Iqbal, 2018). Notably, despite the insignificant difference, the rate of anaemia in α‐tocopherol sufficient group was lower than that of α‐tocopherol deficient group in our study, which suggests a potential functional connection between α‐tocopherol level and haematopoiesis.

Our study has a few limitations. First, the levels of vitamins in a population may be affected by several factors, such as geography, diet, and age. Second, we recruited patients from a single medical site in northern China; therefore, our cohort may not represent a broader pregnant population in the country. Third, the data for retinol and α‐tocopherol were collected from the pregnant population at our institute without applying any exclusion criteria. For example, the pregnancy‐related dieting complications, such as hyperemesis gravidarum, may also affect vitamin intake and absorption and result in decreased levels of vitamins A and E in serum. Multiple pregnancy may also have significant effect on vitamin levels due to their extra needs of nutrients.

In summary, a laboratory‐developed method for quantitation of serum retinol and α‐tocopherol was validated with good performance. With over 31,000 paired retinol and α‐tocopherol measurements, we were able to establish the trimester‐specific RIs with the Hoffmann method. Although no significant changes were noticed in the retinol level during pregnancy, the α‐tocopherol level significantly increased with the increasing gestational age. Simultaneously, the association between retinol levels and anaemia was confirmed. Further, increased retinol and decreased α‐tocopherol were associated with higher PLT counts, thereby implying their different roles in inflammation.

## CONFLICTS OF INTEREST

The authors declare that they have no conflicts of interest.

## CONTRIBUTIONS

JL and ZC designed the study. JL, SZ, YJ, YL, and YL were involved in the vitamin LC‐MS/MS quantitation method development and validation. JL, GT, YD, YZ, and LL extracted, analysed the data, and performed the statistical analysis. JL and ZC wrote the manuscript, and all authors contributed to manuscript revision and approved the final copy.

## Supporting information


**Figure S1.** **A**: The representative quantile‐quantile plot (QQ‐plot) of the retinol level in the first trimester (n = 11429). Theoretical quantiles are deviations from the mean in multiples of the standard deviation of a standard normal distribution (μ = 0, σ = 1; μ represents the mean and σ represents the standard deviation). **B**: The re‐graph of the QQ‐plot of the visually determined linear portion from the Supplementary Figure 1A

## References

[mcn12975-bib-0001] Achebe, M. M. , & Gafter‐Gvili, A. (2017). How I treat anemia in pregnancy: Iron, cobalamin, and folate. Blood, 129(8), 940–949. 10.1182/blood-2016-08-672246 28034892

[mcn12975-bib-0002] Albahrani, A. A. , Rotarou, V. , Roche, P. J. , & Greaves, R. F. (2016). A simultaneous quantitative method for vitamins A, D and E in human serum using liquid chromatography‐tandem mass spectrometry. J Steroid Biochem Mol Biol, 159, 41–53. 10.1016/j.jsbmb.2016.02.019 26924585

[mcn12975-bib-0003] Bastos Maia, S. , Costa Caminha, M. F. , Lins da Silva, S. , Rolland Souza, A. S. , Carvalho Dos Santos, C. , & Batista Filho, M. (2018). The prevalence of vitamin A deficiency and associated factors in pregnant women receiving prenatal care at a reference maternity hospital in Northeastern Brazil. Nutrients, 10(9). 10.3390/nu10091271 PMC6165532 30205601

[mcn12975-bib-0004] Brown, C. C. , & Noelle, R. J. (2015). Seeing through the dark: New insights into the immune regulatory functions of vitamin A. Eur J Immunol, 45(5), 1287–1295. 10.1002/eji.201344398 25808452 PMC4426035

[mcn12975-bib-0005] Cartmel, B. , Moon, T. E. , & Levine, N. (1999). Effects of long‐term intake of retinol on selected clinical and laboratory indexes. Am J Clin Nutr, 69(5), 937–943. 10.1093/ajcn/69.5.937 10232634

[mcn12975-bib-0006] Chen, H. , Qian, N. , Yan, L. , & Jiang, H. (2018). Role of serum vitamin A and E in pregnancy. Exp Ther Med, 16(6), 5185–5189. 10.3892/etm.2018.6830 30542475 PMC6257734

[mcn12975-bib-0007] Chen, Y. J. , Li, Z. D. , Mao, C. Y. , Kang, X. , & Zhang, S. H. (2018). An investigation of the levels of vitamins A, D, and E in the serum of Chinese pregnant women. J Clin Lab Anal, 32(1). 10.1002/jcla.22176 PMC6816855 28220968

[mcn12975-bib-0008] Connell, E. (2012). Tietz textbook of clinical chemistry and molecular diagnostics (5th edn). Human Pathlogy, 49(6), 615–615.

[mcn12975-bib-0009] da Cunha, M. S. B. , Campos Hankins, N. A. , & Arruda, S. F. (2018). Effect of vitamin A supplementation on iron status in humans: A systematic review and meta‐analysis. Crit Rev Food Sci Nutr, 59, 1–15. 10.1080/10408398.2018.1427552 29336593

[mcn12975-bib-0010] Freedman, J. E. , & Keaney, J. F. Jr. (2001). Vitamin E inhibition of platelet aggregation is independent of antioxidant activity. J Nutr, 131(2), 374S–377S. 10.1093/jn/131.2.374S 11160564

[mcn12975-bib-0011] Furr, H. C. (2004). Analysis of retinoids and carotenoids: Problems resolved and unsolved. J Nutr, 134(1), 281S–285S. 10.1093/jn/134.1.281S 14704334

[mcn12975-bib-0012] Graham‐Maar, R. C. , Schall, J. I. , Stettler, N. , Zemel, B. S. , & Stallings, V. A. (2006). Elevated vitamin A intake and serum retinol in preadolescent children with cystic fibrosis. Am J Clin Nutr, 84(1), 174–182. 10.1093/ajcn/84.1.174 16825693

[mcn12975-bib-0013] Grebe, S. K. , & Singh, R. J. (2011). LC‐MS/MS in the clinical laboratory—Where to from here? Clin Biochem Rev, 32(1), 5–31. 21451775 PMC3052391

[mcn12975-bib-0014] Hoffmann, G. , Lichtinghagen, R. , & Wosniok, W. (2016). Simple estimation of reference intervals from routine laboratory data. J Lab Med.. 10.1515/labmed-2015-0104

[mcn12975-bib-0015] Hoffmann, R. G. (1963). Statistics in the practice of medicine. JAMA, 185, 864–873. 10.1001/jama.1963.03060110068020 14043090

[mcn12975-bib-0016] Holmes, D. T. , & Buhr, K. A. (2019). Widespread incorrect implementation of the Hoffmann Method, the correct approach, and modern alternatives. Am J Clin Pathol, 151(3), 328–336. 10.1093/ajcp/aqy149 30475946

[mcn12975-bib-0017] Horowitz, G. L. , Altaie, S. P. , Boyd, J. C. , Ceriotti, F. M. , Garg, U. , Horn, P. , … Zakowski, J. (2010). Defining, establishing, and verifying reference intervals in the clinical laboratory; approved guideline—Third Edition. Clinical and Laboratory Standards Institute document EP28‐A3c, 28(30), 1–59. 10.1016/j.clinbiochem.2014.03.025

[mcn12975-bib-0018] Jia, Y. , Zhan, S. E. , Zhai, Y. H. , Xiao‐Mei, J. I. , Qin, S. Z. , & Zheng, C. (2018). Determination of vitamin A and E in serum by liquid chromatography tandem‐mass spectrometry. Labeled Immunoassays & Clinical Medicine, 25(4), 574–579.

[mcn12975-bib-0019] Jilani, T. , & Iqbal, M. P. (2018). Vitamin E deficiency in South Asian population and the therapeutic use of alpha‐tocopherol (Vitamin E) for correction of anemia. Pak J Med Sci *,* 34(6), 1571‐1575. doi:10.12669/pjms.346.15880 30559825 10.12669/pjms.346.15880 PMC6290196

[mcn12975-bib-0020] Jones, G. R. D. , Haeckel, R. , Loh, T. P. , Sikaris, K. , Streichert, T. , Katayev, A. , … Decision, L. (2018). Indirect methods for reference interval determination—Review and recommendations. Clin Chem Lab Med, 57(1), 20–29. 10.1515/cclm-2018-0073 29672266

[mcn12975-bib-0021] Katayev, A. , Balciza, C. , & Seccombe, D. W. (2010). Establishing reference intervals for clinical laboratory test results: Is there a better way? Am J Clin Pathol, 133(2), 180–186. 10.1309/AJCPN5BMTSF1CDYP 20093226

[mcn12975-bib-0022] Katayev, A. , Fleming, J. K. , Luo, D. , Fisher, A. H. , & Sharp, T. M. (2015). Reference intervals data mining: No longer a probability paper method. Am J Clin Pathol, 143(1), 134–142. 10.1309/AJCPQPRNIB54WFKJ 25511152

[mcn12975-bib-0023] Marchese, M. E. , Kumar, R. , Colangelo, L. A. , Avila, P. C. , Jacobs, D. R. Jr. , Gross, M. , … Cook‐Mills, J. M. (2014). The vitamin E isoforms alpha‐tocopherol and gamma‐tocopherol have opposite associations with spirometric parameters: The CARDIA study. Respir Res, 15, 31 10.1186/1465-9921-15-31 24629024 PMC4003816

[mcn12975-bib-0024] Mcguire, S . (2012). WHO Guideline: Vitamin A supplementation in pregnant women. Geneva: WHO, 2011; WHO Guideline: Vitamin A supplementation in postpartum women. Geneva: WHO, 2011. *Advances in Nutrition,* 3(2), 215. 10.3945/an.111.001701 PMC3648723 22516730

[mcn12975-bib-0025] Midttun, O. , McCann, A. , Aarseth, O. , Krokeide, M. , Kvalheim, G. , Meyer, K. , & Ueland, P. M. (2016). Combined measurement of 6 fat‐soluble vitamins and 26 water‐soluble functional vitamin markers and amino acids in 50 uL of Serum or plasma by high‐throughput mass spectrometry. Anal Chem, 88(21), 10427–10436. 10.1021/acs.analchem.6b02325 27715010

[mcn12975-bib-0026] Murohara, T. , Ikeda, H. , Katoh, A. , Takajo, Y. , Otsuka, Y. , Haramaki, N. , & Imaizumi, T. (2002). Vitamin E inhibits lysophosphatidylcholine‐induced endothelial dysfunction and platelet activation. Antioxid Redox Signal, 4(5), 791–798. 10.1089/152308602760598945 12470507

[mcn12975-bib-0027] Nakano, T. , Hanasaki, K. , Matsumoto, S. , & Arita, H. (1988). Retinol induces platelet aggregation via activation of phospholipase A2. Biochem Biophys Res Commun, 154(3), 1075–1080. 10.1016/0006-291x(88)90250-1 3408485

[mcn12975-bib-0028] Ohrvall, M. , Sundlof, G. , & Vessby, B. (1996). Gamma, but not alpha, tocopherol levels in serum are reduced in coronary heart disease patients. J Intern Med, 239(2), 111–117. 10.1046/j.1365-2796.1996.410753000.x 8568478

[mcn12975-bib-0029] Olmedilla, B. , Granado, F. , Southon, S. , Wright, A. J. , Blanco, I. , Gil‐Martinez, E. , … Thurnham, D. I. (2001). Serum concentrations of carotenoids and vitamins A, E, and C in control subjects from five European countries. Br J Nutr, 85(2), 227–238. 10.1079/bjn2000248 11242491

[mcn12975-bib-0030] Raizman, J. E. , Cohen, A. H. , Teodoro‐Morrison, T. , Wan, B. , Khun‐Chen, M. , Wilkenson, C. , … Adeli, K. (2014). Pediatric reference value distributions for vitamins A and E in the CALIPER cohort and establishment of age‐stratified reference intervals. Clin Biochem, 47(9), 812–815. 10.1016/j.clinbiochem.2014.03.025 24726493

[mcn12975-bib-0031] Rubin, L. P. , Ross, A. C. , Stephensen, C. B. , Bohn, T. , & Tanumihardjo, S. A. (2017). Metabolic effects of inflammation on vitamin A and carotenoids in humans and animal models. Adv Nutr, 8(2), 197–212. 10.3945/an.116.014167 28298266 PMC5347109

[mcn12975-bib-0032] Rumbold, A. , Ota, E. , Hori, H. , Miyazaki, C. , & Crowther, C. A. (2015). Vitamin E supplementation in pregnancy. Cochrane Database Syst Rev, 9, CD004069 10.1002/14651858.CD004069.pub3 PMC8406700 26343254

[mcn12975-bib-0033] Semba, R. D. , & Bloem, M. W. (2002). The anemia of vitamin A deficiency: Epidemiology and pathogenesis. Eur J Clin Nutr, 56(4), 271–281. 10.1038/sj.ejcn.1601320 11965502

[mcn12975-bib-0034] Strukova, S. (2006). Blood coagulation‐dependent inflammation. Coagulation‐dependent inflammation and inflammation‐dependent thrombosis. Front Biosci, 11, 59–80. 10.2741/1780 16146714

[mcn12975-bib-0035] Wagner, K. H. , Kamal‐Eldin, A. , & Elmadfa, I. (2004). Gamma‐tocopherol—An underestimated vitamin? Ann Nutr Metab, 48(3), 169–188. 10.1159/000079555 15256801

[mcn12975-bib-0036] Wang, Y. , Gao, Y. , Liu, Q. , Zhan, X. , Li, Z. , Hu, H. , … Chen, J. (2016). Effect of vitamin A and Zn supplementation on indices of vitamin A status, haemoglobin level and defecation of children with persistent diarrhea. J Clin Biochem Nutr, 59(1), 58–64. 10.3164/jcbn.15-68 27499581 PMC4933690

[mcn12975-bib-0037] West, K. P. Jr. (2002). Extent of vitamin A deficiency among preschool children and women of reproductive age. J Nutr, 132(9 Suppl), 2857S–2866S. 10.1093/jn/132.9.2857S 12221262

[mcn12975-bib-0038] Whitin, J. C. , Gordon, R. K. , Corwin, L. M. , & Simons, E. R. (1982). The effect of vitamin E deficiency on some platelet membrane properties. J Lipid Res, 23(2), 276–282. 7077141

[mcn12975-bib-0039] Yang, C. , Chen, J. , Liu, Z. , Yun, C. , Piao, J. , & Yang, X. (2016). Prevalence and influence factors of vitamin A deficiency of Chinese pregnant women. Nutr J, 15, 12 10.1186/s12937-016-0131-7 26818747 PMC4729160

[mcn12975-bib-0040] Zhang, Z. H. , Ni, M. , & Hu, Y. (2018). Current status of vitamin A deficiency in preschool children in Dongguan, China and the effect of vitamin A on serum ferritin and red blood cell parameters. Zhongguo Dang Dai Er Ke Za Zhi, 20(3), 195–199. 29530118 10.7499/j.issn.1008-8830.2018.03.006 PMC7389791

