# Supplementary figures and images for: Retinol and α‐tocopherol in pregnancy: Establishment of reference intervals and associations with CBC
Source: Matern Child Nutr. 2020 Mar 5;16(3):e12975. doi: 10.1111/mcn.12975 (PMC7296784; doi:10.1111/mcn.12975)

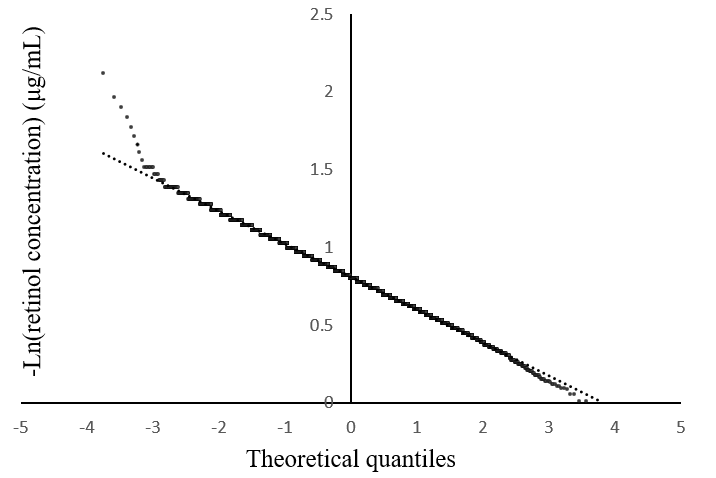

Supplement: Supplementary file 1 — Figure S1. A: The representative quantile‐quantile plot (QQ‐plot) of the retinol level in the first trimester (n = 11429). Theoretical quantiles are deviations from the mean in multiples of the standard deviation of a standard normal distribution (μ = 0, σ = 1; μ represents the mean and σ represents the standard deviation). B: The re‐graph of the QQ‐plot of the visually determined linear portion from the Supplementary Figure 1A [file MCN-16-e12975-s001.tif]
